# The US President's Cancer Panel: A Model For Gathering Country-Level Input to Inform Cancer Control Policy and Program Initiatives

**DOI:** 10.1200/GO.22.00410

**Published:** 2023-03-15

**Authors:** Lisa J. Paradis, Ann Chao, Barbara K. Rimer, Abby B. Sandler, Kalina Duncan, Mishka Kohli Cira, Rachel Hanisch

**Affiliations:** ^1^ Former Employee of US National Cancer Institute (2011-2018), Rockville, MD; ^2^ US National Cancer Institute, Rockville, MD; ^3^ UNC Gillings School of Global Public Health, Chapel Hill, NC

## Abstract

**PURPOSE:**

The President's Cancer Panel (Panel) is a federal advisory committee charged with monitoring the US National Cancer Program and reporting directly to the US President. Since its creation a half century ago, the Panel has gathered input from individuals and organizations across the US cancer community and beyond and recommended actions to accelerate progress against cancer. The Panel is unique in its structure and function, and merits examination for its potential applicability in other settings worldwide.

**METHODS:**

We present an overview of the general President's Cancer Panel model and describe the noteworthy and unique characteristics of the Panel that help achieve its charge. We also detail the specific processes, outputs, and achievements of the Panel appointed by President Barack Obama, which served between 2012 and 2018.

**RESULTS:**

From 2012 to 2018, the Panel focused on three topics that addressed timely issues in cancer prevention and control: (1) HPV vaccination for cancer prevention, (2) connected health and cancer, and (3) value and affordability of cancer drug treatment. The Panel held 11 meetings with 165 participants who provided diverse perspectives on these issues. Four reports were delivered to the president, which were cited about 270 times in the literature. Over 20 collaborator activities, including commitments of funding, can be linked to the recommendations published in these reports.

**CONCLUSION:**

The US President's Cancer Panel highlights the importance of independent advisory bodies within a national cancer control program and of national leadership support for the cancer community. The structure and function of the Panel could be applicable in other settings worldwide.

## INTRODUCTION

Landmark legislation signed 50 years ago in the United States marked an amplification in how cancers are studied and treated, and how research is translated and applied. The National Cancer Act of 1971 (P.L. 92-218) was signed into law in December 1971, after years of heightened national attention on cancer as an urgent public health threat.^1-4^ Mobilizing support for cancer around the theme of a War on Cancer was an important part of achieving bipartisan support for greatly enhanced funding for cancer.

**CONTEXT**

**Key Objective**
What potential role can an independent advisory body play in advancing the objectives of a national cancer control program? The US President's Cancer Panel, which advises the US President on issues of urgent importance to the national cancer community, is an example of potential relevance to the global cancer landscape.
**Knowledge Generated**
The US President's Cancer Panel provides a unique model for engaging high-profile members of the cancer community to make timely, actionable, and evidence-based recommendations to national leaders to address the burden of cancer. Knowledge of the Panel's history, processes, activities, and lessons learned can be applied in other settings worldwide to help ensure that cancer prevention and control is a priority of public health planning efforts.
**Relevance**
The US President's Cancer Panel advisory model may be relevant and applicable in settings worldwide where regional or national cancer control programming is a planned or desired priority.

**FIG 1:**
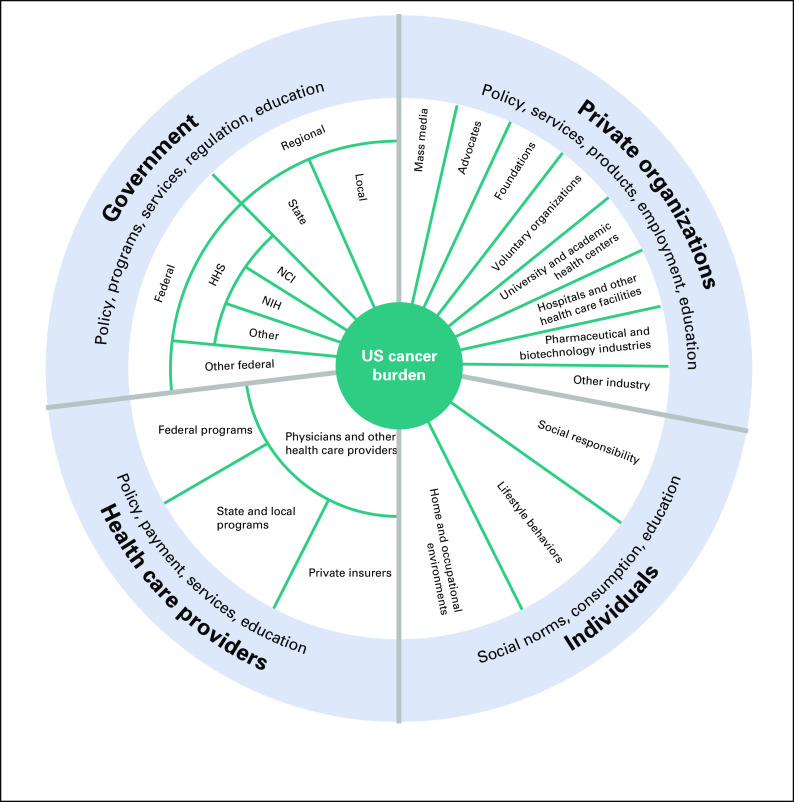
The US National Cancer Program. Adapted from the study by Reuben et al.^3^

Among the key pioneering efforts of the Act was the establishment of the President's Cancer Panel (the Panel), a federal advisory committee that monitors the execution of the activities of the National Cancer Program (NCP), a broad, complex collection of governmental and nongovernmental agencies, organizations, activities, and programs that play a role in addressing the US cancer burden (Fig 1).^3,5,6^

### Overview of the US President's Cancer Panel

The architects of the 1971 National Cancer Act acknowledged the important role that input from individuals outside the federal government could play when setting NCP priorities. The Act established numerous committees to provide such direction. One of these, the President's Cancer Panel, reports directly to the US President. The Act noted that members “by virtue of their training, experience, and background [be] exceptionally qualified to appraise the NCP.”^4^

The Panel is a federal advisory committee governed by the Federal Advisory Committee Act (FACA) of 1972 [P.L. 92-463], which was established to ensure that bodies advising the federal government provide objective guidance that is accessible to the public.^7^ Operations of the Panel are supported by the National Cancer Institute (NCI), an agency within the National Institutes of Health (NIH), under the cabinet-level agency, the US Department of Health and Human Services. To our knowledge, the Panel is the only NIH FACA committee that advises the President directly rather than the leadership of various NIH institutes.^8^ This is consistent with the NCI's special budgetary authority and reporting relationship.

The Panel accomplishes its mission by working with interested parties to identify issues of critical importance to the NCP and national cancer community and by developing reports to the President with high-level program and policy recommendations. The President and White House advisors are the primary audience for the Panel's reports, but the Panel's work is also intended to support federal, state, and nongovernmental organizations in addressing public health priorities highlighted within. Once Panel reports are published, implementation of recommendations is the responsibility of those with the authority to implement them, as the Panel is strictly an advisory body.

Since inception, Panels have produced reports to the President on a range of topics such as cancer survivorship, accelerating scientific innovation, and environmental factors associated with cancer. Reports are available online from 1996.^9^

### The 2012-2018 US President's Cancer Panel

In this report, we present a brief overview of the President's Cancer Panel as a model and highlight the processes, outputs, and achievements of the Panel appointed by President Barack Obama, which served between 2012 and 2018 (the Obama Panel). This Panel comprised chair B.K.R. (DrPH, a cancer control researcher), Hill Harper (JD, activist, cancer survivor, and actor), and Owen N. Witte (MD, a clinical translational physician-researcher).^10,11^ We also describe some important features of the Panel and note lessons learned from the Obama Panel's experiences. We also present examples of other US and international advisory bodies on cancer. We maintain that the Panel's unique function merits examination for its potential applicability in other settings worldwide.

## METHODS

Methods used for this report included describing the key legislation that led to formation of the President's Cancer Panel, an overview of the Panel in general, and a description of the Obama Panel in greater detail. Three authors (R.H., L.J.P., and A.B.S.) were staff members for the Obama Panel, and one author (Barbara K. Rimer) was the Panel Chair. These authors reviewed and documented records of Panel activities, work processes, outputs, and achievements.

As a secondary study aim, we assessed the number of times Panel reports have been cited in the published literature, from the time of each report's release to January 24, 2022. The databases Scopus (Elsevier) and Google Scholar were searched using search terms that included “President's Cancer Panel” and variations of the title of each Panel report. The full citation of all search results was then exported to EndNote 20; duplicates were identified and removed. All citations were reviewed for applicability and accuracy; one error (published in 2006) was removed. The search included English language literature only without other exclusion criteria. This citation search was conducted to quantify the number of times that each Panel report was referenced in the published literature as a surrogate measure of the potential reach of each report; it was not a systematic literature search or scoping review.

## RESULTS

The National Cancer Act called on the Panel to review the NCP by holding periodic public hearings and submitting an annual report directly to the President.^4^ Notably, it did not include specific language on how the Panel should conduct operations. This autonomy has enabled Panels over the years to have flexibility about how to focus their annual reviews of the NCP. In this section, we describe how President Obama's Panel conducted its activities, which followed the general Panel model but also introduced unique activities into panel proceedings, such as topic selection criteria (Table 1) and involvement of panel cochairs and collaborators in early phases of recommendation and report development. We also detail the reports this Panel published, provide citations of these reports in the literature as described above in the Methods section, and describe known subsequent actions taken by the cancer community.

**TABLE 1:**
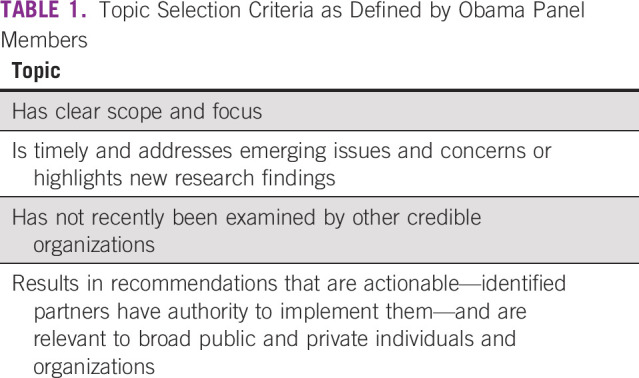
Topic Selection Criteria as Defined by Obama Panel Members

### Key Process Steps

For each report, the Panel developed a concept for a topic of interest, identified key individuals and held meetings to gather input, conducted additional secondary research, synthesized meeting findings, and drafted recommendations (Table 2). Each Panel report was delivered to the White House and released to the public in print and digital formats.

**TABLE 2:**
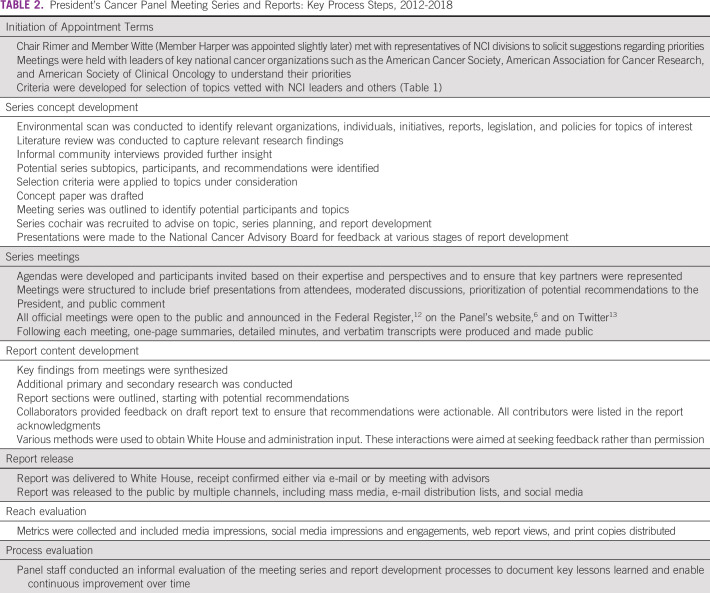
President's Cancer Panel Meeting Series and Reports: Key Process Steps, 2012-2018

### Meeting Series

From 2012 to 2018, the Obama Panel focused on three topics that addressed important and timely issues in cancer prevention and control: (1) human papillomavirus (HPV) vaccination for cancer prevention, (2) connected health and cancer, and (3) value and affordability of cancer drug treatment (Table 3). Each series included numerous invited organizations and individuals, and all meetings were open to the public and provided opportunity for public comment. Although the primary focus of each report was the United States, the Panel considered global implications in its reports on HPV vaccination.

**TABLE 3:**
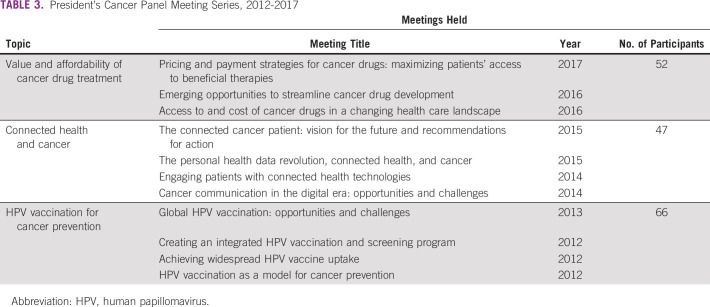
President's Cancer Panel Meeting Series, 2012-2017

### Report Audience and Reach

The primary audience for Panel reports is the US President along with relevant White House advisors. Secondary audiences include other stakeholders in the NCP such as federal agencies, professional and voluntary health organizations, and members of the public. Producing print and digital forms of the reports enabled use of multiple channels to disseminate the reports, such as live events, e-mail and social media, and mass media. Digital delivery made the reports much more accessible to a wider audience, searchable, and usable.

Table 4 presents the four reports produced by the Obama Panel and citations of each report found in the literature. One or more of the four reports was cited in the literature 270 times, with the largest number of citations referring to the report on HPV vaccination published in 2014. In total, 261 citations were journal articles, four were books/electronic books, two were conference proceedings, and two were serials. Articles citing Panel reports were featured in a wide range of public health, cancer-specific, and topic-specific journals (eg, health informatics, women's health, and health communication) and national and organizational progress reports and websites (Data Supplement).

**TABLE 4:**
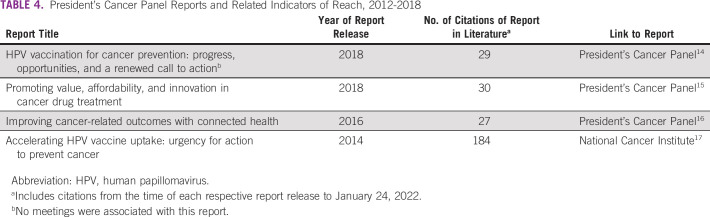
President's Cancer Panel Reports and Related Indicators of Reach, 2012-2018

### Related Collaborator Actions

As the Panel is an advisory body, it can call on individuals and organizations in the field to implement its recommendations. The Obama panel engaged potential implementers in the entire report process so they would be more likely to act on recommendations, and this was an effective strategy. Partners made commitments they might not have made had they not been involved from the beginning of each Panel report process.

Noteworthy actions that we identified are summarized in Table 5

**TABLE 5:**
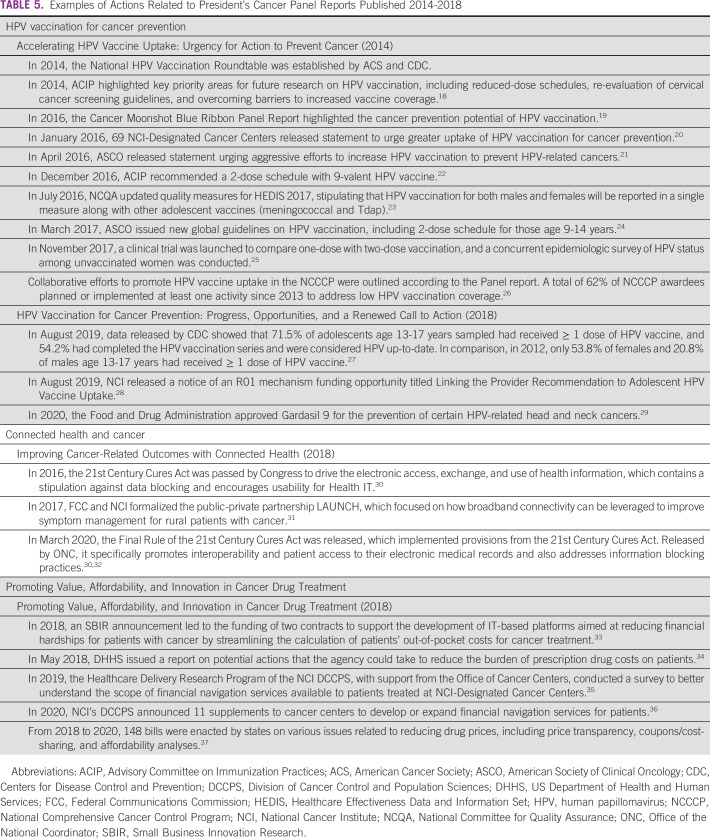
Examples of Actions Related to President's Cancer Panel Reports Published 2014-2018

. Some activities can be attributed directly to Panel recommendations. These include seeding collaborations, such as the National HPV Vaccination Roundtable, hosted by the American Cancer Society and funded in part by the Centers for Disease Control and Prevention (CDC), and the formation of the NCI and Federal Communications Commission's public-private partnership called LAUNCH (Linking and Accelerating User-Centered Networks through Connected Health).

Numerous other actions are aligned closely with Panel recommendations, although they did not explicitly refer to Panel reports. These include NCI funding opportunity announcements to NCI-designated cancer centers to support HPV vaccination and to develop or expand financial navigation services for patients.^36^

## DISCUSSION

Since its creation a half century ago, the President's Cancer Panel has served as a model for elevating cancer-related issues facing the nation, and recommendations to address them, directly to the US President. The ways in which panels achieved this aim have evolved over time. In this section, we describe noteworthy characteristics of the panel model in recent decades and highlight specific lessons learned from the experiences of the Obama Panel. This exercise may be helpful in examining the potential utility and application of an advisory body like the President's Cancer Panel in other settings globally.

Important features of the Panel as a model include a lean and agile structure, authority, autonomy, convening power, priority-setting ability, and practical relevance to the national cancer community. These characteristics have helped the Panel examine priority topics and provide recommendations to address national cancer challenges in a structured and efficient manner while achieving support and visibility for its reports.

The Panel model offers an efficient structure, comprising a small group of renowned experts and staff. In general, Panel members are appointed to serve for three-year terms with the possibility of reappointments and extension. Two of the three members are distinguished physicians or scientists.^38^ They are designated as Special Government Employees of the US federal government and must undergo ethics training and complete a financial disclosure before finalizing their appointments.^39,40^ Members are not compensated full time but rather on the occasions when they are actively participating in Panel-related activities. The Panel is supported by NCI staff members who are federal employees or contractors.

Collaborators are engaged to extend the capabilities and subject matter expertise of the Panel. These individuals aid the Panel in a consultative manner; government allowances are provided for costs of travel to meetings. As such, they are not permanently on staff, which supports innovation and agility and flexibility in operations. Examples of active partner involvement in Panel activities include suggesting meeting participants, participation in Panel meetings, contribution to consensus-building and priority-setting activities, and involvement in report drafting via directed feedback.

Being associated with the President of the United States gives the Panel's recommendations high visibility, authority, and wide recognition, potentially lending credibility to the Panel's recommendations. The Panel also has the authority to convene meetings that are considered official proceedings of the US government. Meetings are listed in the Federal Register, and proceedings become part of the official federal record.

The Panel also enjoys a certain level of autonomy in its ability to achieve its mission. This priority-setting ability supports the development of recommendations that are focused, specific, and—ultimately—actionable.

Although the primary audience of its reports is the President, the Panel maximizes the utility of its work by reaching a broader audience, including US government executive branch agencies, professional organizations, policymakers, states, patients, advocates, and the general public. These audiences are taken into consideration when developing final reports, and the Panel has in recent decades engaged the media and interested parties to promote each report's release. The Panel also has an open participation policy; the public is invited to provide testimony for the official record. Patients and their advocates are included for participation in Panel meetings.

Although the unique operating model of the President's Cancer Panel in recent decades supports the execution of its mission, it is associated with some challenges. These include issues related to the role of the Panel as an advisory but not an implementation body, the broad scope of its mission, and its susceptibility to a varied US political landscape. The Obama Panel took steps—some of which are detailed below—to minimize the impact of these challenges on its work.

The Panel does not have the authority to implement its recommendations. However, the Obama Panel made concerted efforts to engage partners that had implementation authority and worked closely with these groups. For its 2012-2013 report on accelerating HPV vaccine uptake, the Panel engaged the CDC and American Cancer Society early in the series-planning activities for direction. It also engaged immunization partners, which were generally not associated with cancer prevention and control activities at the time. Bringing together diverse partners who were not previously at the same table ensured that the recommendations were appropriate and actionable.

The charge of the Panel, as defined in the National Cancer Act, is to monitor the NCP, a considerable charge given the size and scope of the program, particularly for a small advisory committee. Indeed, past Panels have called for an evaluation of the NCP to clarify its mission and vision, enhance coordination, and identify accountable parties.^3^

Over many years, the Panel has generally approached this challenge by selecting one area of focus per series or report. The Obama Panel took extra steps to define and apply a set of criteria to potential topics. Focusing areas of study is nonetheless challenging. For example, the Obama Panel set out to examine the changing landscape in cancer communications in 2014-2015, but it was difficult to narrow this subject to a meaningful and manageable topic. The Panel went on to develop a series and report on connected health and cancer.^16^ The time spent working to understand this topic area and the needs of communities resulted in alignment on other high-profile efforts in 2016, including the Cancer Moonshot Taskforce^41^ and the 21st Century Cures Act.^42,32^

Unless the Panel is directly credited and cited, it is difficult to directly attribute actions to the Panel's recommendations. Also, although not unique to the Panel as a FACA committee, the Panel has lacked a formal tracking and evaluation system for its work, output, and impact. For the purposes of this report, a citation search of literature using recognized search engines was undertaken. This search found that the Obama Panel reports have been cited by others who are publishing work in the areas that the Panel studied. Panel reports served several key objectives in these articles, including providing calls to action and recommendations that align with those of authors. Panel reports, extensively referenced themselves, also helped expand the evidence base provided in these articles. These citation search results indicate a wide breadth and utility of Panel reports as support for authors' published work. However, while informative, these results cannot be characterized as a formal tracking of the impact of the Panel's work.

A formal tracking strategy and support for this activity would help to better evaluate the impact of the Panel's activities and recommendations on cancer research, care, and policy initiatives. Partly with this challenge in mind, in 2018, the Obama Panel revisited HPV vaccination 5 years after the publication of its initial report. In a report from the Panel chair, the HPV landscape was re-examined to understand where needs remained and to issue an updated call to action to increase HPV vaccination rates globally.^14^ This report was not a formal evaluation, but it provided a unique opportunity to assess the progress made and re-energize efforts in areas where work was still needed.

Although designed to have three members serving overlapping 3-year terms to maximize continuity, the Panel is vulnerable to disruption in operations. When one administration leaves and a new one comes in, Panel members may potentially step down and leave a gap until new members are appointed. The process involved with onboarding a new Panel member may also cause disruptions to Panel operations because many of the decisions made by the three-member Panel require a quorum. Fortunately, Panel members may serve until replaced if they are willing and able, which has helped minimize gaps in operations.

Other national groups in the United States act in an advisory capacity on cancer.^43-45^ The National Cancer Advisory Board was established by the National Cancer Act of 1971, like the Panel.^43^ The National Cancer Advisory Board contains a mix of presidential appointees and nonvoting ex officio members. Its charge is to advise the NCI director on NCP activities, including research priorities, training, and grants. The National Cancer Policy Forum, part of the National Academies of Science, Engineering, and Medicine, examines issues of interest to the broad national and international cancer communities.^44^ Like the Panel, the National Cancer Policy Forum convenes workshops and produces reports that identify critical actions that can be taken to reduce the burden of cancer.

Several global examples of groups acting in an advisory capacity on cancer also exist. One of the recognizable characteristics of the US President's Cancer Panel is its intrinsic link to the President of the United States. In France, a similar history of political leaders championing national cancer control efforts can also be tracked, beginning with President Jacques Chirac's signing of the Charter of Paris 2000 to the 2021-2030 cancer control strategy unveiled by President Emmanuel Macron that is implemented by the French National Cancer Institute.^46,47^ In Taiwan, the Cancer Control Act of 2003 led to the establishment of the Cancer Prevention and Control Policy Commission, which formulates cancer prevention and control policy, evaluates the cancer control budget, and reviews matters such as guidelines for diagnosis and treatment.^48^ The All-Party Parliamentary Group on Cancer (APPGC) in the United Kingdom also has a similar priority-setting charge as the Panel. The APPGC was founded in 1998 to keep cancer at the top of the political agenda, and to ensure that policymaking remains patient centered.^49^ APPGC officers meet regularly throughout the year, make decisions on activity and policy direction for the group, and serve as champions for cancer care in Parliament. As part of these regular meetings, APPGC also discusses priorities and hears from expert speakers on topics of interest.

In conclusion, the US President's Cancer Panel, in operation for 50 years, issues recommendations on high-priority topics to monitor the execution of the National Cancer Program. Features of the Panel have helped maximize these efforts, including the Panel's structure, authority, priority-setting ability, and reach. The Panel highlights the importance of independent advisory bodies within a national cancer control program and of national leadership support for the cancer community. The unique structure and function of the President's Cancer Panel could be applicable in other settings worldwide.

## References

[1] Rettig RA Cancer Crusade: The Story of the National Cancer Act of 1971 Lincoln, NE iUniverse 2005

[2] Weinhouse S National Cancer Act of 1971—An Editorial Cancer Res 32 i ii 1972 5014797

[3] Reuben S Milliken E Paradis L for the President's Cancer Panel The Future of Cancer Research Accelerating Scientific Innovation Bethesda, MD 2012

[4] National Cancer Institute The National Cancer Act. Cancer.gov 2012 https://www.cancer.gov/about-nci/overview/history/national-cancer-act-1971#cancer-program

[5] Rauscher FJ Research and the National Cancer Program Science 189 115 119 1975 1138368 10.1126/science.1138368

[6] President’s Cancer Panel | Advisors to the President on the National Cancer Program https://prescancerpanel.cancer.gov/

[7] US General Services Administration Federal Advisory Committee Act (FACA) Management Overview https://www.gsa.gov/policy-regulations/policy/federal-advisory-committee-act-faca-management-overview

[8] National Institutes of Health Office of Federal Advisory Committee Policy—NIH Committees https://ofacp.od.nih.gov/committees/index.asp

[9] President’s Cancer Panel Reports https://deainfo.nci.nih.gov/advisory/pcp/annualReports/index.htm

[10] The White House Office of the Press Secretary. President Obama Announces More Key Administration Posts. whitehouse.gov 2011 https://obamawhitehouse.archives.gov/the-press-office/2011/11/29/president-obama-announces-more-key-administration-posts

[11] The White House Office of the Press Secretary. President Obama Announces More Key Administration Posts. whitehouse.gov 2012 https://obamawhitehouse.archives.gov/the-press-office/2012/08/01/president-obama-announces-more-key-administration-posts

[12] Federal Register The Daily Journal of the United States Government. Federal Register https://www.federalregister.gov/

[13] President's Cancer Panel (@PresCancerPanel)/Twitter. https://twitter.com/prescancerpanel

[14] President’s Cancer Panel HPV Vaccination for Cancer Prevention: Progress, Opportunities, and a Renewed Call to Action 2018 https://prescancerpanel.cancer.gov/report/hpvupdate/

[15] President’s Cancer Panel Promoting Value, Affordability, and Innovation in Cancer Drug Treatment, 2018 https://prescancerpanel.cancer.gov/report/drugvalue/

[16] President’s Cancer Panel Improving Cancer-Related Outcomes with Connected Health 2016 https://prescancerpanel.cancer.gov/report/connectedhealth/

[17] Accelerating HPV Vaccine Uptake: Urgency for Action to Prevent Cancer, 2014 https://deainfo.nci.nih.gov/advisory/pcp/annualReports/HPV/index.htm

[18] Markowitz LE Dunne EF Saraiya M et al Human papillomavirus vaccination: Recommendations of the Advisory Committee on Immunization Practices (ACIP) MMWR Recomm Rep 63 1 30 2014 25167164

[19] National Cancer Institute Cancer Moonshot^SM^ Blue Ribbon Panel 2016 https://www.cancer.gov/research/key-initiatives/moonshot-cancer-initiative/blue-ribbon-panel

[20] NCI-Designated Cancer Centers Urge HPV Vaccination for the Prevention of Cancer https://healthcaredelivery.cancer.gov/hpvuptake/nci_hpv_consensus_statement_012716.pdf

[21] Bailey HH Chuang LT duPont NC et al American Society of Clinical Oncology statement: Human papillomavirus vaccination for cancer prevention J Clin Oncol 34 1803 1812 2016 27069078 10.1200/JCO.2016.67.2014

[22] Meites E Kempe A Markowitz LE Use of a 2-dose schedule for human papillomavirus vaccination—Updated recommendations of the Advisory Committee on Immunization Practices MMWR Morb Mortal Wkly Rep 65 1405 1408 2016 27977643 10.15585/mmwr.mm6549a5

[23] National Committee for Quality Assurance (NCQA) NCQA Updates Quality Measures For HEDIS® 2017 https://www.ncqa.org/news/ncqa-updates-quality-measures-for-hedis-2017/

[24] Arrossi S Temin S Garland S et al Primary prevention of cervical cancer: American Society of Clinical Oncology resource-stratified guideline JCO Glob Oncol 3 611 634 2017 10.1200/JGO.2016.008151 PMC5646902 29094100

[25] Kreimer AR Rodriguez AC Hildesheim A et al Proof-of-principle evaluation of the efficacy of fewer than three doses of a bivalent HPV16/18 vaccine J Natl Cancer Inst 103 1444 1451 2011 21908768 10.1093/jnci/djr319 PMC3186781

[26] Townsend JS Steele CB Hayes N et al Human papillomavirus vaccine as an anticancer vaccine: Collaborative efforts to promote human papillomavirus vaccine in the National Comprehensive Cancer Control Program J Women's Health 26 200 206 2017 10.1089/jwh.2017.6351 PMC5774011 28263672

[27] Elam-Evans LD Yankey D Singleton JA et al. National, regional, state, and selected local area vaccination coverage among adolescents aged 13–17 years—United States, 2019 MMWR Morb Mortal Wkly Rep 69 1109 1116 2020 32817598 10.15585/mmwr.mm6933a1 PMC7439984

[28] Department of Health and Human Services PAR-19-360: Linking the Provider Recommendation to Adolescent HPV Vaccine Uptake (R01 Clinical Trial Optional) https://grants.nih.gov/grants/guide/pa-files/par-19-360.html

[29] GARDASIL 9. FDA 2020 https://www.fda.gov/vaccines-blood-biologics/vaccines/gardasil-9

[30] National Archives Federal Register: 21st Century Cures Act: Interoperability, Information Blocking, and the ONC Health IT Certification Program https://www.federalregister.gov/documents/2020/05/01/2020-07419/21st-century-cures-act-interoperability-information-blocking-and-the-onc-health-it-certification

[31] Federal Communications Commission FCC-NCI Broadband Cancer Collaboration 2017 https://www.fcc.gov/health/cancer

[32] Sweeney Anthony E T he Cures Act Final Rule: Interoperability-Focused Policies that Empower Patients and Support Providers. Health IT Buzz: The Latest on Health Information Technology from ONC 2020 https://www.healthit.gov/buzz-blog/21st-century-cures-act/the-cures-final-rule

[33] National Cancer Institute NIH/NCI 384—Digital Healthcare Platform to Reduce Financial Hardship for Cancer Patients | NCI: SBIR & STTR https://sbir.cancer.gov/funding/contracts/384

[34] US Department of Health & Human Services American Patients First: The Trump Administration Blueprint to Lower Drug Prices and Reduce Out-of-Pocket Costs 2018 https://www.hhs.gov/sites/default/files/AmericanPatientsFirst.pdf

[35] de Moor JS Mollica M Sampson A et al Delivery of financial navigation services within national cancer institute-designated cancer centers JNCI Cancer Spectr 5:pkab033 2021 10.1093/jncics/pkab033 PMC8242138 34222790

[36] National Cancer Institute Financial Hardship During Cancer Treatment | Division of Cancer Control and Population Sciences (DCCPS) https://cancercontrol.cancer.gov/research-emphasis/financial-hardship

[37] State Drug Pricing Laws: 2017-2021—The National Academy for State Health Policy https://www.nashp.org/rx-laws/

[38] National Cancer Institute President’s Cancer Panel Charter Summary https://deainfo.nci.nih.gov/advisory/pcp/charterSummary.pdf

[39] US General Services Administration Special Government Employees (SGE) https://www.gsa.gov/policy-regulations/policy/federal-advisory-committee-management/advice-and-guidance/special-government-employees-sge

[40] National Institutes of Health Ethics Training for Special Government Employees https://ethics.od.nih.gov/ethics-training-special-government-employees

[41] The White House Office of the Press Secretary. The Vice President’s Cancer Moonshot https://obamawhitehouse.archives.gov/node/352601

[42] National Institutes of Health The 21st Century Cures Act https://www.nih.gov/research-training/medical-research-initiatives/cures

[43] National Cancer Institute National Cancer Advisory Board https://deainfo.nci.nih.gov/advisory/ncab/ncab.htm

[44] National Cancer Policy Forum | National Academies https://www.nationalacademies.org/our-work/national-cancer-policy-forum

[45] Welcome to the CEO Roundtable on Cancer | CEO Roundtable on Cancer https://www.ceoroundtableoncancer.org/

[46] World Cancer Day Celebrating 20 Years of World Cancer Day https://www.worldcancerday.org/20thanniversary

[47] The French National Cancer Institute www.en.ecancer.fr https://en.e-cancer.fr/

[48] Research Office, Legislative Council Secretariat Cancer Strategy in Taiwan 2019 https://www.legco.gov.hk/research-publications/english/1819fs07-cancer-strategy-in-taiwan-20190624-e.pdf

[49] Macmillan Cancer Support All-Party Parliamentary Group on Cancer https://www.macmillan.org.uk/about-us/what-we-do/we-make-change-happen/we-work-with-governments/appgc

